# 

**Published:** 1868-01

**Authors:** 


### INDEX OF HALL'S JOURNAL OF HEALTH.

### VOL. XV, 1868.

| | PAGE |
|---|---|
| Annoyances | 163 |
| Brain and Body | 41 |
| Bronchitis | 67, 135 |
| Benevolence | 83 |
| Bed, Costly | 116 |
| Bed-Chambers | 180 |
| Boxing Ears | 217 |
| Bed-Rooms | 260 |
| Buckwheat Cakes | 287 |
| Bathing | 289 |
| Colds Prevented | 37, 56 |
| Costivness | 55 |
| Catching Cold | 58 |
| Comfort | 62, 114 |
| Central Park | 81 |
| Clerical Longevity | 100 |
| Cough, Stomach | 159 |
| Consumption, Contagious | 169 |
| Cellars | 178 |
| Catarrh | 189 |
| Cold Water at Meals | 226 |
| Coffee | 236 |
| Crazy Man | 265 |
| Dentistry | 46 |
| Debt and Disease | 97 |
| Disease from Dirt | 206 |
| Disease Threatened | 283 |
| Daughters Ruined | 288 |
| Eating Wisely | 57 |
| Eating by Rule | 59 |
| Early Rising | 64 |
| Egotism | 160 |
| Eyesight | 218 |
| Eggs, Keeping | 244 |
| Fire on the Hearth | 49 |
| Feet, Attention to | 54 |
| French Cookery | 93 |
| Food and Drink | 225 |
| Food Cure | 251 |
| Face Painting | 264 |
| Going to the Country | 129 |
| Grandmother Howell | 157 |
| Getting Sick | 161 |
| Guillotine | 215 |
| Glass of Brandy | 274 |
| Husbands and Wives | 96 |
| Hard Times | 201 |
| Hobby Horsed | 221 |
| Healthful Localities | 254 |
| Healthful Atmosphere | 257 |
| House Building | 258 |
| Hay Fever | 266 |
| Human Health | 278 |
| Ingrowing Toe Nail | 96 |
| Idleness | 100 |
| Ice Water | 173 |
| Intemperance | 185, 186, 200, 202 |
| Insanity | 208 |
| Kindness Rewarded | 294 |
| Lungs Strengthened | 44 |
| Life Lengthened | 61, 66 |
| Life's Discipline | 63 |
| Living Long | 91 |
| Living by Rule | 134 |
| Life a Probation | 192 |
| Linen | 222 |
| Lunacy | 223 |
| Lifting Cure | 261 |
| Memory Impaired | 17, 282 |
| Marriage Maxims | 33, 99, 252 |
| Mental Rest | 39 |
| May Moving | 78 |
| Madness | 98 |
| Manners, Engaging | 99 |
| Mind and Charcoal | 121 |
| Mad Dog | 126, 199 |
| Medicine, Taking | 198 |
| Maine Law | 230 |
| Maccaroni | 271 |
| Men Wanted | 273 |
| Non Sequitur | 164 |
| Night Air | 256 |
| New Ideas | 290 |
| Old School House | 157 |

| | PAGE. | | PAGE. |
|---|---|---|---|
| Onions | 259 | Spitting Blood | 179 |
| Poverty and Longevity | 66 | Sun Baths | 249 |
| Presentiments | 77 | Saving Life | 253 |
| Potatoes | 95, 237 | Sea Sickness | 262 |
| Power, its Source | 121 | Suicide | 270 |
| Pulse | 132 | Simples | 280 |
| Popular Errors | 207 | Sound Sleep | 281 |
| Pickles | 238 | Small Pox | 291 |
| Preserving | 238 | Typhoid Fever | 60 |
| Principles, Fixed | 276 | Traveling for Health | 101 |
| Physician, Good | 288 | Table Manners | 170 |
| Revaccination | 127 | Tomatoes | 172, 240 |
| Recognition Hereafter | 224 | Tobacco Users | 177 |
| Regimen | 174 | Teeth | 216, 275 |
| Reforms | 229 | Tea and Coffee | 228, 235 |
| Schools, Public | 4 | Toast | 240 |
| Skating | 19 | Taking the Papers | 284 |
| Stones | 50 | World's Worthies | 273 |
| Salt of the Earth | 65 | Warming Dwellings | 31, 165 |
| Smoking | 100, 209 | Water Drinking | 184, 255 |
| Summering | 112 | Write, When to | 214 |
| Starvation | 135 | Youth, Dangers of | 183 |
| Sleeping | 175 | Youth and Age | 195 |
| Succeed, How to | 181, 201 | Ventilation | 162, 166 |

### MONTHLY PUBLICATIONS.

Atlantic Monthly, devoted to Literature, Science, Art and Politics, published by Ticknor & Fields, 124 Tremont Street, Boston, Mass.; office for New York and Brooklyn subscribers, 63 Bleecker Street, New York, a few doors east of Broadway; — $4 a year, single numbers 35 cents.

American Agriculturist, $1 50 a year, 41 Park Row, New York, pub lished by Orange Judd & Co., devoted to all that pertains to the farm, garden and orchard.

Arthur's Magazine, $2. a year, 811 Chestnut Street, Philadelphia.

Blackwood's Magazine, or any one of the Reviews, $4; with any one Review, $7. The 4 Reviews, $12. Blackwood and the 4 Reviews, 15.00. 38 Walker Street, west of Broadway. The Leonard Scott Publishing Company, New York.

Beadle's Monthly; A Magazine of To-Day. $3 a year; 118 William Street, near Fulton. American News Co., Agents, 119 and 121 Nassau Street, New York.

Family Treasure; A Religious and Literary Monthly, W. T. Findley, D. D., Editor and Publisher. $2. Three copies to one address, $5. 65 W. 4th Street, Cincinnati, Ohio.

Gardeners' Monthly and Horticultural Advertiser, edited by Thomas Meehan, No. 23 North South Street, Philadelphia. $2 a year.

Godey's Ladies' Book, Vol. 75, edited by Mrs. Sarah J. Hale and L. A. Godey, $3 a year, corner Sixth and Chestnut Streets, Philadelphia, with numerous and beautiful embellishments each month, Fashion plates, Patterns, &c., &c.

Harper's Weekly, $4 a year, in advance, single copies 10 cents. Harper's Magazine, published monthly at Four Dollars a year; postage 24 cents a year; on the Weekly, 20 cents. 331 Pearl Street, New York.

Horticulturist, and Journal of Rural Art and Rural Taste, published at $2.50 a year, or 25 cents singly, by Woodward, 37 Park Row, New York. Established in 1846.

Hours at Home, $1.25, published by T. S. Arthur & Co., Philadelphia.

Half-Yearly Abstract of The Medical Sciences, being an Analytical and Critical digest of the principal British and Continental Medical works in the preceeding six months, by Henry C. Lea, Philadephia. $2.50 a year, single numbers $1.50

Maryland Farmer, devoted to Agriculture, Horticulture, Rural Economy and Mechanic Arts, published by S. Sands, Mills & Co. at $1.50 a year. 21 South Calbert Street, Baltimore, Md.

Merry's Museum, $1.50 a year, 172 William Street, New York.

Mother's Journal and Family Visitant, edited by Miss Caroline O. Hiscox and published by Sheldon & Company, 500 Broadway, New York.

New England Farmer, published by Eaton & Co., 34 Merchants Row, Boston. $1.50 a year, single copies 15 cents; 52 pages, 8vo, monthly.

Sabbath at Home, an illustrated religious magazine for the family, published by the American Tract Society, 28 Cornhill, Boston, and at 13 Bible House, New York, by the Messrs. Broughton & Wyman. $2 a year, or $2 50.for six months.

